# Expectations in Romantic Relations and Psychological Well-Being of Adolescents in Pakistan: Moderating Role of Parental Support

**DOI:** 10.5334/pb.556

**Published:** 2021-03-10

**Authors:** Sofia T. Cheema, Jamil A. Malik

**Affiliations:** 1 National Institute of Psychology, Quaid-i-Azam University, Islamabad, PK

**Keywords:** Expectations, Romantic relations, Adolescents, Perceived parental support, Psychological well-being

## Abstract

The objective of the current study was to investigate the role of perceived parental support as a moderator in the association between adolescents’ expectations in romantic relations and their psychological well-being. The sample consisted of 647 adolescents (boys = 285, girls = 362). Their age ranged from 16 to 18 years (*M* = 17.19 years, *SD* = .77) and they were regular students in different colleges of Islamabad and Rawalpindi. They completed the Perceived Parental Support Scale, the Well-being Questionnaire-W-BQ12 and the Romantic Relations Scale for Adolescents. The results showed that there were significant gender differences on expectations in romantic relations and psychological well-being with girls scoring higher than boys on expectations in romantic relations while boys scoring higher than girls on psychological well-being. The results also indicated that there was a significant negative association between expectations in romantic relations and psychological well-being. Findings of the moderation analysis showed that perceived parental support moderated the association. Simple slope analysis indicated that there was a significant negative slope for low and medium levels of perceived parental support while the slope was non-significant for high levels of perceived parental support. These results indicated that perceived parental support counters the negative effect of expectations in romantic relations on psychological well-being during adolescence. It is suggested that perceived parental support is important in planning interventions to improve the well-being of adolescents.

## Expectations in Romantic Relations and Psychological Well-Being of Adolescents in Pakistan: Moderating Role of Parental Support

Romantic relations can develop at any stage of life, but they are usually considered as hallmark of adolescence (Giordano et al., 2006). Although, once adolescents’ romantic relationships were considered trivial, superficial, transitory and as a product of social dysfunction, now they are considered important or significant for their development (Collins, 2003; Collins et al., 2009; Furman & Shaffer, 2003; Furman & Collins, 2008). Recent studies have shown that romantic relationships have significance for the well-being of the adolescents (Collins, 2003; Collins et al., 2009; Giordano et al., 2006). These relationships have not only mental health benefits for adolescents but also provide them social support, improve their self-esteem, prepare them for adult relationships and develop intimacy (Collins, 2003; Connolly & Goldberg, 1999).

As the aforementioned literature shows the importance of romantic relations in the lives of adolescents, it also reported the existence of expectations in romantic relations. According to Burgoon (1995), expectations are a natural phenomenon, and in every culture, people have certain expectations about others’ behaviour. In romantic relations, people also have certain expectations regarding romantic partner’s behaviour. When people are in love, they expect that their partner will take them as a special person, will take care of them, will have time for them, will give them gifts and will help them in case of problems (Kokab & Ajmal, 2012). Literature also shows that in romantic relations, there are expectations of social companionship, emotional closeness, relationship positivity (Fuhrman et al., 2009), pleasant personality and physical attractiveness (Eggermont, 2004). In a qualitative study conducted in Pakistan, it was found that adolescent girls had variant expectations in romantic relations. The study found that the girls expect love, care, attention and time or companionship from their boyfriends while the boys expect time or companionship and sincerity from their girlfriends. Furthermore, the boys also expected that their girlfriend will give them importance (Cheema & Malik, 2021). Literature also shows that there are expectations of having sexual relations as reported by Brown et al. (1999) stating that there are expectations of eventually, if not now, having sexual relations in romantic relationships.

Although in the west, people accept the romantic relationships of adolescents and consider these relations important in the development of adolescents (Collins, 2003; Collins et al., 2009; Furman & Shaffer, 2003; Furman & Collins, 2008), in non-western cultures like Pakistan, the situation is quite different. Although attraction for opposite gender individuals is normal but it is not accepted (Ali, 2011). The majority of the population is Muslim and premarital relationships are not allowed in Islam hence romantic relations are considered “*haram*” (forbidden). Additionally, in the prevailing collectivist culture, family is given importance and adolescents have lesser autonomy for taking decisions related to the choice of life partner leaving no room for acceptance of romantic relations. Although there is a scarcity of literature on the romantic relations of adolescents and nothing can be said about the prevalence of romantic relations of adolescents living in Pakistan, it cannot be denied that adolescents living in Pakistan have a concept of romantic relations as discussed in a qualitative study conducted by Cheema and Malik (2021).

The literature evidenced the existence of expectations in romantic relations (Brown et al., 1999; Cheema & Malik, 2021; Eggermont, 2004; Fuhrman et al., 2009; Kokab & Ajmal, 2012) and the association of adolescents’ romantic relations with their well-being (Collins, 2003; Collins et al., 2009; Giordano et al., 2006) but the question that how the adolescents’ expectations in romantic relations are related to their psychological well-being remained unexplored. As Pakistan is a religious, collectivist and conventional society where relationships between opposite gender individuals are not accepted or appreciated (Ali, 2011), it can be assumed that expectations regarding romantic relations are discouraged. Hence, it can be assumed that the romantic relationships or expectations regarding romantic relationships will have negative effect on the psychological well-being of adolescents. In the present study, it was hypothesized that expectations in romantic relations and psychological well-being of the adolescents have a negative association.

Love and romantic relations may become important during adolescence. On the other hand, family, especially parents, and peers remain the main sources of support for adolescents. Research on adolescents showed that parents remained a major source of support for adolescents even when the peer support became more important for them (Youniss & Smollar, 1985) and often parental support remained important throughout their college years (Furman & Wehner, 1997). Parker and Benson (2004) found that the high parental monitoring and parental support during adolescence were related to high self-esteem. It has also been found that parental support and friends’ support is related to greater well-being and social adjustment (Lee & Goldstein, 2015). In a study conducted by Hussey et al. (2013), the researchers found that parental support was an important predictor of psychological well-being of the adolescents, both male and female. Parental support is not only related to psychological well-being of the adolescents, but it is also related to their social adjustment and well-being in social relations. It fosters adjustment and well-being in social relationships, particularly in romantic relationships (Lee et al. 2015; Mounts et al., 2006).

Like all other relationships, romantic relationships are also rooted in social networks hence their functioning is influenced by their social network members e.g., by friends and parents (Felmlee, 2001). Friends’ and parents’ positive opinion about romantic relations and their support is very important for adolescents and found to be related to the initiation and maintenance of these relationships (Etcheverry et al., 2008). Acceptance of romantic relationships by these social networks leads to their support (Etcheverry & Agnew, 2004). Sprecher and Felmlee (1992, 2000) found in their longitudinal study that perceived support from family and friends predicted commitment and satisfaction. Parents’ support promotes the maintenance of romantic relationship and absence of their support may lead to the end of these relationships. The aim of present study was to test the direct effect of parental support on psychological well-being of adolescents in Pakistan. Further it was hypothesized that parental support moderates the relationship between expectations in romantic relations and psychological well-being.

Following the literature, the present study conceptualised a moderation model. It is hypothesized that expectations in romantic relations have a negative association with psychological well-being of adolescents, whereas perceived parental support has a positive association with psychological well-being. Further it is hypothesized that parental support acts as a moderator to counter the negative impact of expectations in romantic relation on the psychological well-being of adolescents.

## Method

### Instruments

#### Romantic Relations Scale for Adolescents (RRS-A)

To measure the adolescents’ perception of expectations in romantic relations, the Romantic Relations Scale for Adolescents (RRS-A), developed by the authors of this study, was used. The scale has three-dimensions i.e., Intimacy, Passion and Distrust. The Intimacy dimension has seven factors including Sincerity, Expectations, Sharing, Closeness, Understanding, Pleasure and Significance. The Passion dimension consists of three factors including Motive to love, Physical Attraction, and Companionship. While the Distrust dimension has three factors namely Disloyalty, Negative Dating Attitude, and Lack of Commitment. For the present study only the expectations factor of the Intimacy dimension was used. It measures adolescents’ perception of expectations of love, care, attention, trust, understanding and sincerity in a romantic relationship. It has six items with six response categories that range from *completely disagree* (0) to *completely agree* (5). Scores on all items are summed up to get the total score that ranges from 0 to 30. The total score of the Expectations factor was taken as an indicator of the adolescents’ perception of expectations in romantic relations. The higher the score the higher the perception of expectations in romantic relations. Its Cronbach’s Alpha coefficient was .71.

#### Well-Being Questionnaire-W-BQ12

Psychological Well-being of adolescents was measured by using the 12-item Well-being Questionnaire (Bradley, 2000) that consisted of three sub-scales i.e., Negative Well-being (4 items), Energy (4 items) and Positive Well-being (4 items). The scores of three sub-scales were summed up to get the psychological well-being score. A higher score on this questionnaire showed a higher level of psychological well-being. It is a four-point rating scale with a Cronbach’s Alpha coefficient of .87 (Sagduyu et al., 2003). In the present study, the Urdu version of W-BQ12 was used.

#### Perceived Parental Support Scale-PPSS

Perceived parental support was assessed by using the Perceived Parental Support Scale-PPSS. The scale measures the adolescents’ perception of their accessibility to general support from their parents (Kristjansson et al., 2011). It is a five-item scale having four response categories which are *very difficult* (1), *rather difficult* (2), *rather easy* (3) and *very easy* (4). The score on the scale ranges from 5 to 20. A higher score on this scale indicates a higher perception of parental support. Cronbach’s Alpha coefficient was found to vary from .77 to .87 in different studies (Kristjansson et al., 2010; Kristjansson et al., 2011). In order to administer the scale in Urdu language, the scale was translated and adapted by the authors of the present study by using the “Forward translation and back translation” method.

### Sample

A sample of 647 adolescents (boys = 285, girls = 362) was taken from different public (*n* = 416) and private (*n* = 231) colleges of Islamabad and Rawalpindi. Participants included students of the 11^th^ grade (*n* = 361) and the 12^th^ grade (*n* = 286). The age range of the students was 16 to 18 years (*M* = 17.19 years, *SD* = .77). Family system distribution showed that 33.7% of the adolescents were coming from a joint family system, a family system where three generations i.e., grandparents, parents and grandchildren were living together (Akhtar et al., 2017), while 66.3% were from a nuclear family system. Adolescents whose fathers were self-employed constituted 36.5% of the sample, adolescents having fathers who were employed in public sector composed 35.5% of the sample, fathers of 23.6% adolescents were employed in private sector whereas fathers of remaining 3.4% adolescents had been retired from different organizations. Mothers of the majority of the sample i.e., 93% were housewives and only 4.6% mothers of the adolescents were employees in a public sector whereas 1.1% adolescents have mothers employed in a private sector. The remaining 1.2% adolescents’ mothers were self-employed.

### Procedure

After receiving formal permission from the principals/directors of colleges and parents of the students and informed consent of the adolescents, scales/questionnaires were administered. Only those adolescents who were students of the 11^th^ or 12^th^ grade in a public or private college and of whom both of the parents were alive were included in the study. They were informed about the purpose of the study and they were assured that all information taken from them would be used only for this particular research and would be kept confidential. Written as well as verbal instructions were provided by the researcher of this study. All the participants completed scales/questionnaires during their college time, in the presence of the researcher of the study. They took approximately 15 to 20 minutes to complete the measures. After data collection, scores were calculated, and data was analysed.

### Analysis

Data was analysed using IBM-SPSS version 21. Descriptive statistics of the study variables and Pearson correlations between all variables i.e., study variables and demographic variables, were computed as a preliminary analysis. To investigate gender differences in the study variables, *t*-tests were used. Finally, moderation analysis was conducted to test the moderation hypothesis. Moderation was tested using the Process Macro (Hayes, 2013).

## Results

Descriptive statistics of the study variables such as mean, standard deviation, Cronbach’s Alpha, range, skewness, and kurtosis were calculated which are displayed in ***Table 1***. Skewness and kurtosis values are in the acceptable range. The Cronbach’s Alpha coefficients of the PPSS, W-BQ12 and the Expectations factor of the Intimacy sub-scale of RRS-A are .73, .74 and .75, respectively.

**Table 1:** Descriptive Statistics for study variables (*N* = 647).


VARIABLES	NO. OF ITEMS	*M*	*SD*	CRONBACH’S ALPHA	RANGE		SKEWNESS	KURTOSIS

					POTENTIAL	ACTUAL		

Expectations	6	25.56	4.59	.75	0–30	4.98–30	–1.79	4.24

Parental Support	5	16.44	2.93	.73	5–20	5–20	–.93	.58

Psychological Well-being	12	23.33	6.06	.74	0–36	3–36	–.39	.01


The results of Pearson correlations between all variables i.e., study variables and demographic variables (See ***Table 2***), indicate that there is a significant negative correlation between perception of expectations in romantic relations and psychological well-being of the adolescents (*r* = –.12, *p* < .01) and a significant positive correlation between perceived parental support and psychological well-being (*r* = .31, *p* < .01). But the correlation between perception of expectations in romantic relations and perceived parental support is nonsignificant. For demographic variables, age is found to be positively correlated with expectations (*r* = .13, *p* < .01) while father’s and mother’s education are found to be positively correlated with perceived parental support (*r* = .16, *p* < .01 for father’s education, *r* = .21, *p* < .01 for mother’s education). To rule out the influence of these variables, they were controlled for the moderation analysis.

**Table 2:** Correlation matrix among demographic and study variables (*N* = 647).


VARIABLES	1	2	3	4	5	6	7

1. Age	–	–.16**	–.16**	–.08*	.13**	–.06	–.05

2. Father’s education *(FYS)*		–	.56**	.37**	.04	.16**	.02

3. Mother’s education *(FYS)*			–	.25**	.04	.21**	.02

4. Monthly income				–	–.01	.01	–.00

5. Expectations					–	–.04	–.12**

6. Parental support						–	.31**

7. Psychological Well-being							–


* *p* < .05, ** *p <* .01, *FYS* = formal years of schooling.

The results of *t*-tests show that there are significant gender differences on expectations (*t* = –3.17, *p* < .01) and psychological well-being (*t* = 4.71, *p* < .01) while no gender differences are found on perceived parental support (See ***Table 3***). The results indicate that girls scored higher than boys on expectations while boys scored higher than girls on psychological well-being.

**Table 3:** Mean, Standard deviation and t-values for boys and girls on study variables (*N* = 647).


	BOYS (*n* = 285)		GIRLS (*n* = 362)		*t* (645)	*p*	95% *CI*		COHEN’S *d*
		
	*M*	*SD*	*M*	*SD*			*LL*	*UL*	

Parental Support	16.50	2.80	16.40	3.03	.44	.66	–.35	.56	.03

Expectations	24.81	4.26	26.14	4.76	–3.71	.00	–2.04	–.63	.29

Psychological Well-being	24.58	5.73	22.35	6.13	4.71	.00	1.30	3.14	.38


Moderation analysis was conducted by controlling age, father’s education, mother’s education, and monthly income (for moderation analysis see ***Table 4***). Results indicate that perceived parental support significantly moderates the association between adolescents’ expectations in romantic relations and their psychological well-being (*B-*interaction = .04, *p* < .05).

**Table 4:** Showing role of Perceived Parental Support as moderator on association between Expectations in romantic relations and Psychological Well-being (*N* = 647).


PREDICTORS	PSYCHOLOGICAL WELL-BEING		

	*B*	95% *CI*	

		*LL*	*UL*

Constant	27.55**	17.17	37.92

Age	–.21	–.80	.37

Father’s education	–.03	–.16	.11

Mother’s education	–.04	–.15	.07

Monthly income	.02	–.10	.13

Expectations	–.16**	–.26	–.06

Parental Support	.63**	.47	.79

Expectations*Parental Support	.04*	.01	.08

*R^2^*	.12		

*F*	12.14**		


* p < .05, ** p < .01.

**Figure 1:**
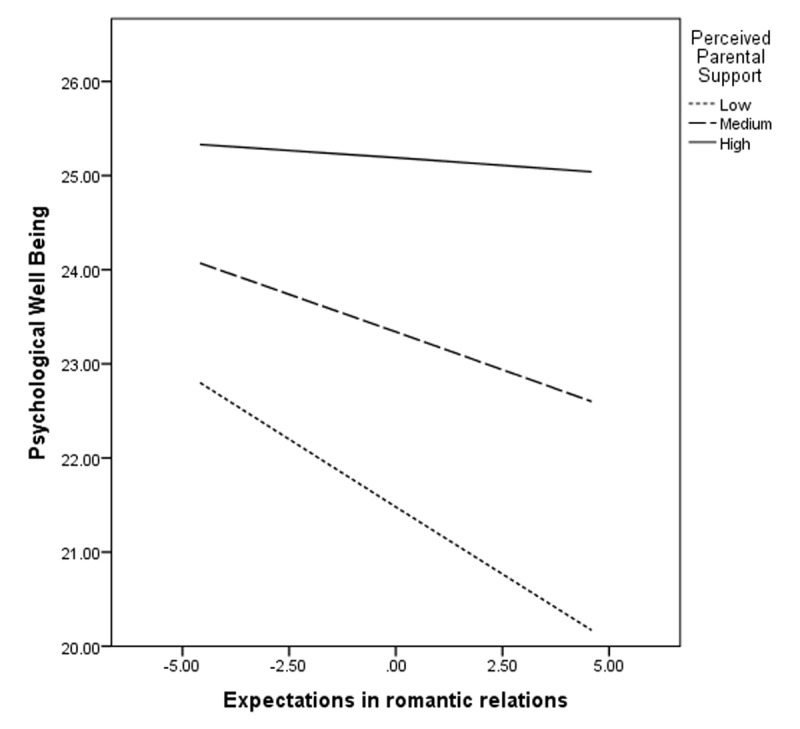
Showing role of Perceived Parental Support as moderator on association between Expectations in romantic relations and Psychological Well-being.

The moderation model explains a total of 12% variance (*R^2^* = .12) in psychological well-being. The moderation graph (***Figure 1***) shows that the slopes for low level of perceived parental support (*B* = –.29, *p* < .01) and medium level of perceived parental support (*B* = –.16, *p* < .01) are significant while the slope for high level of perceived parental support (*B* = –.03, *p* > .05) is non-significant.

## Discussion

Expectations are universal and people in all cultures have certain expectations when they interact with other people (Burgoon, 1995). It is very rare to interact with other people without having some expectations regarding their behaviour (Miller & Turnbull, 1986). Like all other human interactions and relationships, there are certain expectations in romantic relations. Previous literature shows that in romantic relations, there are expectations of care, love, attention, companionship, sincerity (Cheema & Malik, 2021), social companionship, emotional closeness, relationship positivity (Fuhrman et al., 2009), pleasant personality, physical attractiveness (Eggermont, 2004), and sexual relations (Brown et al., 1999). In the current study, expectations in romantic relations were considered as a main predictor of psychological well-being of adolescents. The study was designed to investigate the negative association between expectations in romantic relations and psychological well-being and also to test the moderating role of perceived parental support in this association.

The results of both correlation analysis and moderation analysis showed that there was a negative association between expectations in romantic relations and psychological well-being of the adolescents. It means that adolescents who had high expectations in romantic relations, reported low psychological well-being and vice versa. Although, no previous research has investigated the association of expectations in romantic relation of adolescents and their psychological well-being, the association of adolescents’ romantic relations with their psychological well-being has been studied. Previous literature shows that the romantic relationships have significance for the well-being of adolescents (Collins, 2003; Collins et al., 2009; Giordano et al., 2006). And although, there is limited research on the romantic relationship of adolescents and their well-being, these studies suggest that there is a positive correlation (Demir, 2008). In this study, the negative association between expectations in romantic relations and psychological well-being may be due to the fact that Pakistani society is a conventional society where romantic relationships between opposite gender individuals are not publicly accepted and appreciated. Although the attraction between opposite gender individuals is natural, it has no acceptance in Pakistan (Ali, 2011). As most of the people in Pakistan have a religious orientation, and the Islam does not allow romantic relations, so individuals may have negative views about these relations. They like the concept of romantic relations and romantic love in literature, songs, and movies but they are not ready to accept adolescents’ romantic relations due to their religious and social values. Although, literature seems silent about occurrence of romantic relations of adolescents in Pakistan, in a recent study conducted in Pakistan (Cheema & Malik, 2021), it was found that adolescent girls not only have a clear perception of romantic relations, but they also have some expectations in romantic relations. And despite the fact that norms of society are against romantic relations or love affairs, people still have these relations (Kokab & Ajmal, 2012). As romantic relations are not accepted in the religious collectivist culture of Pakistan so adolescents’ expectations in romantic relations negatively influence their psychological well-being.

This study also explored the effect of perceived parental support on psychological well-being. The results of both correlation analysis and moderation analysis showed that perceived parental support had a positive association with psychological well-being. It means that adolescents who have high perceived parental support score are also high on psychological well-being and vice versa. Our results are in line with previous literature which shows that parental support is an important predictor of psychological well-being of the adolescents (Hussey et al., 2013). Literature also supports that parental support promotes adjustment and well-being in social relationships, especially in romantic relationships (Lee et al., 2015; Mounts et al., 2006).

Finally, the findings of the moderation analysis indicated that the perceived parental support significantly moderated the negative association between adolescents’ expectations in romantic relations and their psychological well-being. Simple slope analysis indicated that there was a significant negative slope for adolescents with low or medium level of perceived parental support while the slope was non-significant for adolescents with high level of perceived parental support. These results indicated that high perceived parental support could counter the negative association of expectations in romantic relations and psychological well-being in adolescents. The declining steepness in pattern of slopes showed that the negative relationship between expectations in romantic relations and psychological well-being decreased with increasing parental support. It means parental support acts as an effective support system against the negative impact of expectations in romantic relations on psychological well-being of the adolescents.

The results of correlation analysis and moderation analysis clearly show that perceived parental support is very important for the adolescents living in a religious collectivist society of Pakistan. As they are dependent on their parents, hence parents’ support has great meaning for them. It does not only directly affect their psychological well-being but also moderates the negative association of expectations in romantic relations and psychological well-being. The results of *t*-test analysis show that there was no significant difference between boys and girls on perceived parental support. It means parental support is equally important for adolescent boys and girls living in a religious collectivist society as they are totally dependent on their parents for their financial and emotional needs.

### Limitations and Suggestions

The present study has some limitations. First, the study was cross-sectional in nature, longitudinal studies are needed to understand the temporal associations of the variables over time. Second, the perceived parental support scale, used in this study, measures the perception of general support provided by the parents. In future studies, we can also assess parental support for romantic relations. Third, only the adolescents who were 16 to 18 years old were included in the sample that is a very limited age group. In future studies age range should be expanded.

### Implications

This study will provide a theoretical base for future studies in which these factors will be included. It will also help to understand the effect of expectations in romantic relations and perceived parental support on psychological well-being of adolescents.

This study will also help parents and professionals to understand that as the adolescent’ romantic relations are not the normative and acceptable interpersonal relations in a religious collectivist society, these relations and expectations in these relations are natural and part of adolescent’s normal development and influence the psychological well-being of adolescents. As findings of this study show that parental support can moderate the negative association of expectations in romantic relations and psychological well-being, professionals should include parents if they plan some intervention to improve adolescents’ well-being.
